# Reliability and Accuracy of Inpatient Teledermatology in Asian Patients

**DOI:** 10.1177/26924366251380372

**Published:** 2025-09-17

**Authors:** Martin Man Ho Chung, Ngan Ming Lau, Judy Tze Lei Sham, Ka Chun Cheng, Chi Keung Yeung, Henry Hin Lee Chan, Sze Man Wong

**Affiliations:** ^1^ Division of Dermatology, Department of Medicine, Queen Mary Hospital, Hong Kong, Hong Kong.; ^2^ Division of Dermatology, Department of Medicine, School of Clinical Medicine, The University of Hong Kong, Hong Kong, Hong Kong.

**Keywords:** dermatology, inpatient, teledermatology, telehealth, telemedicine

## Abstract

**Background:**

Previous studies demonstrated the reliability and accuracy of teledermatology in outpatient settings. It is cost effective and can enhance accessibility to specialist care, leading to better patient outcomes. Few studies have investigated inpatient teledermatology, and local data are lacking. This study aimed to determine whether store-and-forward teledermatology is accurate and reliable for inpatient dermatology consultations in Asians.

**Methods:**

During the study period from April to June 2022, consecutive inpatient dermatology consultations were collected at a tertiary referral center in Hong Kong. For each recruited patient, after assessment by an in-house dermatologist, another dermatologist who would practice store-and-forward teledermatology would assess the same patient separately. The primary outcome measured was the concordance of the preferred diagnoses. Factors affecting concordance were evaluated.

**Results:**

A total of 190 Asian patients were recruited. The concordance between the teledermatologist and inpatient dermatologists was 80% for the preferred diagnosis, with a kappa coefficient of 0.75 (95% confidence interval (CI) 0.71–0.78). The concordance of differential diagnoses was 97.4%, with a kappa coefficient of 0.76 (95% CI 0.73–0.8). The diagnostic accuracy rates were 68.8% (53/77) for inpatient dermatologists and 59.7% (46/77) for the teledermatologist. Better quality of clinical photographs (*p* < 0.001) was associated with agreement on the preferred diagnosis.

**Discussion:**

Inpatient teledermatology in real-world settings has been found to be reliable and accurate in Asians. Its utilization should be encouraged to enhance specialist accessibility and improve patient care.

## Introduction

Teledermatology refers to the remote assessment and care of patients with dermatological conditions through telecommunications.^1^ Its importance has been increasingly recognized during the COVID-19 pandemic.^2–4^ It can be particularly useful in isolation facilities, prisons, and hospitals with limited access to dermatology specialty services.^5,6^

There are two common ways to implement telemedicine. The store-and-forward, or asynchronous, method involves accessing the stored patient data for consultation. Live interactive, or synchronous, methods assess patients in real time through virtual methods, such as video conferencing.^7^ The diagnostic process in dermatology relies heavily on the visual assessment of presentations. Therefore, it is an ideal specialty for telemedicine because presenting complaints can be captured through photos or videos.

Teledermatology studies were first reported in the early 1990s, with a steadily growing number of articles published over the last 20 years.^8^ Numerous studies have analyzed the effectiveness of teledermatology, with the majority focusing on outpatient services. Systematic reviews have shown that the store-and-forward method of teledermatology is highly consistent with face-to-face assessment.^8,9^ Recognizing the reliability of teledermatology in outpatient settings and the importance of inpatient dermatology,^10–12^ this study aimed to investigate the reliability and accuracy of inpatient teledermatology. Only a few studies have investigated inpatient teledermatology, and none have been conducted in the Chinese population. Differences in skin color may influence diagnostic accuracy and study results. The disease spectrum also varies between inpatient and outpatient settings, with inpatients more frequently presenting with generalized eruptions and drug-related conditions. In addition, critically ill or frail patients may not permit full or standardized imaging, which can limit diagnostic precision. We hypothesized that the concordance of the initial diagnosis between teledermatologists and inpatient dermatologists while assessing inpatient dermatology consultations in Hong Kong would be high.

## Methods

### Subject recruitment

Consecutive inpatient dermatology consultations were collected during the three-month period from April 1 to June 30, 2022, in a tertiary referral hospital in Hong Kong.

All consecutive inpatient dermatology consultations of adults aged ≥18 years received during the recruitment period, which were available for face-to-face assessment, were included. Verbal consent was obtained for obtaining clinical photographs. Clinical photographs must be available for inclusion in the study. Duplicate consultations of the same patient for review were excluded. Patients with pediatric consultations were excluded.

The minimum value for the Cohen’s kappa coefficient to be expected (i.e., Null hypothesis) is 0.5, taking reference from the study performed by Keller et al.^13^, which was the resultant concordance between non-dermatologists and in-house dermatologist. When the power and alpha were pre-specified at 80.0% and 0.05, respectively, a minimum sample size of 24 was required for the detection of a minimum value of kappa coefficient of 0.8 (near-perfect agreement). We then multiplied the minimum sample size by two, as suggested by the guideline,^14^ to take into account that, in reality, the frequency of each category (diagnosis from each dermatologist) might not be proportional to each other. According to the guideline, the minimum sample size should be smaller for more categorical items, for example, 24 diagnostic categories in the study by Keller et al.^13^; therefore, the minimal required recruitment would be less than 48 in this study.

According to the inpatient dermatology consultation data in our hospital from 2019 to 2020, there were approximately 100 consultations per month. Our recruitment period was set to three months, and the approximate sample for screening would be 300, which was well above the minimal sample size to detect a significant outcome.

Each study subject was assessed separately by an in-house dermatologist as usual clinical service and another dermatologist who would practice store-and-forward teledermatology, i.e., teledermatologist. Clinical photos were taken without flash by the referring parent team using an Apple™ iPad (5th generation) equipped with an 8-megapixel camera and subsequently uploaded without compression for evaluation by teledermatologists. This should be performed on the first day of referral. Adequate lighting and exposure to body parts were emphasized. Two registered dermatology specialists were involved in the study, one of whom reviewed the referral letters and clinical photos of the cases evaluated during inpatient consultations. Clinical photos were reviewed on devices with identical specifications and could be zoomed in and adjusted as needed. The first teledermatologist involved in the study had 8 years of experience in inpatient dermatology, while the other teledermatologist had 10 years of experience in inpatient dermatology. No communication between the inpatient dermatologist and the teledermatologist was allowed. The dermatologists involved would fill in a questionnaire (Supplementary Data S1) to suggest the most likely diagnosis, differential diagnoses, whether a skin biopsy was needed, and the follow-up plan. The teledermatologist also commented on the quality of the clinical photos. The final diagnosis was reviewed after the investigation results were available, including skin biopsy and microbiological workups. The diagnostic accuracy of both the groups was determined. Moreover, the provisional diagnosis by the referring physician was recorded and compared with that suggested by dermatologists.

The primary outcome was concordance of the preferred diagnosis made by the inpatient dermatologist and teledermatologist. Secondary outcomes included concordance in skin biopsy and follow-up decisions, concordance between the referring physicians and dermatologists, accuracy of the diagnosis in each arm, and factors affecting the concordance of the preferred diagnosis. The concordance of the differential diagnoses was defined as the matching of at least one of the differential diagnoses listed.

This study was conducted in compliance with the Declaration of Helsinki and ICH-GCP guidelines and was approved by the institutional review board of the University of Hong Kong/Hospital Authority Hong Kong West Cluster (HKU/HA HKW IRB) (IRB reference number: UW 22-140). All clinical data and photos were kept confidential, and recruited patients were de-identified for data interpretation. Any copy of the data was properly destroyed within three years after the completion of the study.

### Analysis

The concordance stated in the primary and secondary outcomes was assessed using the proportion of agreement and Cohen’s Kappa statistic. The kappa coefficient was interpreted as recommended. 0–0.20 as slight, 0.21–0.40 as fair, 0.41–0.60 as moderate, 0.61–0.80 as substantial, and 0.81–1 as almost perfect agreement, respectively.^15^ Categorical variables were compared using Fisher’s exact test or Pearson’s chi-squared test, as appropriate. The Mann–Whitney *U* test was used to compare the continuous variables. Factors associated with concordance and accuracy were evaluated using univariate and multivariate logistic regression models. Data analysis was performed using IBM SPSS Statistics, version 28.0.1.

## Results

**FIG. 1.**
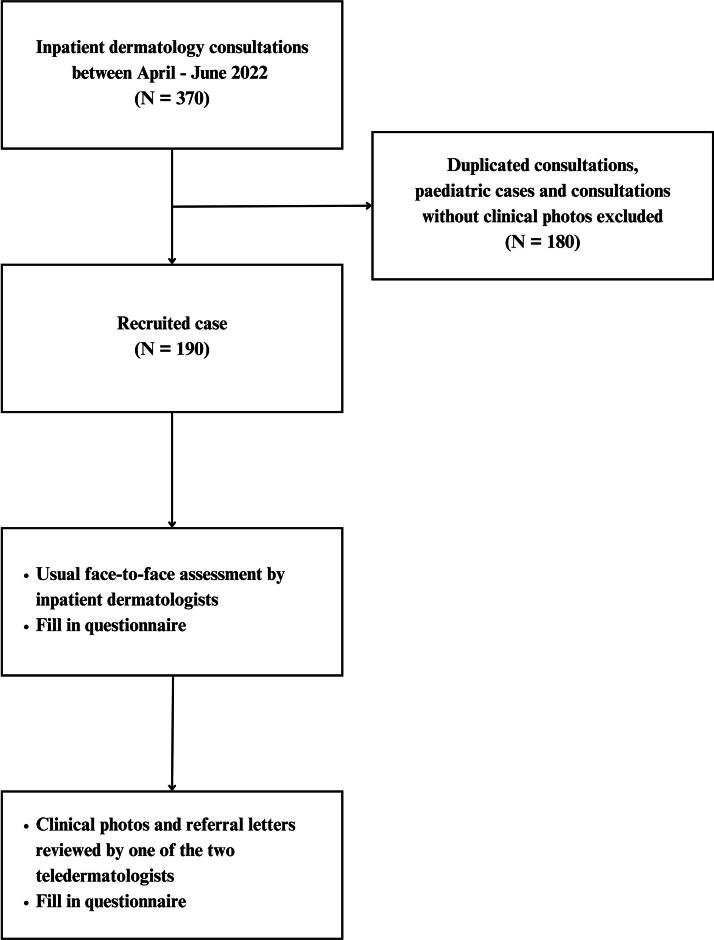
Study design and subject recruitment.

A total of 370 inpatient dermatology consultations were conducted during the study period. Pediatric cases and consultations without clinical photographs were excluded. Duplicated consultations or consultations issued for review were excluded. A total of 190 patients were recruited (Fig. 1).

The mean age was 64.1 years (age range, 19–102 years). A total of 185 (97.4%) patients were Chinese, and 5 (2.6%) patients were of other ethnicities. The sex ratio was equal: 95 (50%) were male and 95 (50%) were female.

The largest source of referral was the Department of Medicine, with a total number of 145 (76.3%) eligible consultations. Of these consultations, 31 (16.3%) were from medicine specialty wards. The second largest source of referral was from the orthopedics ward, with 23 (12.1%) consultations. The number of consultations from the Department of Surgery was 14 (7.4%). The other consultations were from the Obstetrics and Gynecology, Clinical Oncology, and Psychiatry wards.

Approximately half of the referrals (51.6%) did not specify a preliminary diagnosis. The top three most common reasons were nonspecific rash (30%) and cutaneous infections (17.4%), including cellulitis and blistering diseases (12.7%). Eczema (4.2%), dermatitis (3.7%), psoriasis (3.2%), drug eruptions (3.7%), and anticancer drug-related cutaneous eruptions (2.6%) were the other common reasons for consultation. Only two referrals specified a suspected malignancy as a preliminary diagnosis.

### Primary outcome

**FIG. 2.**
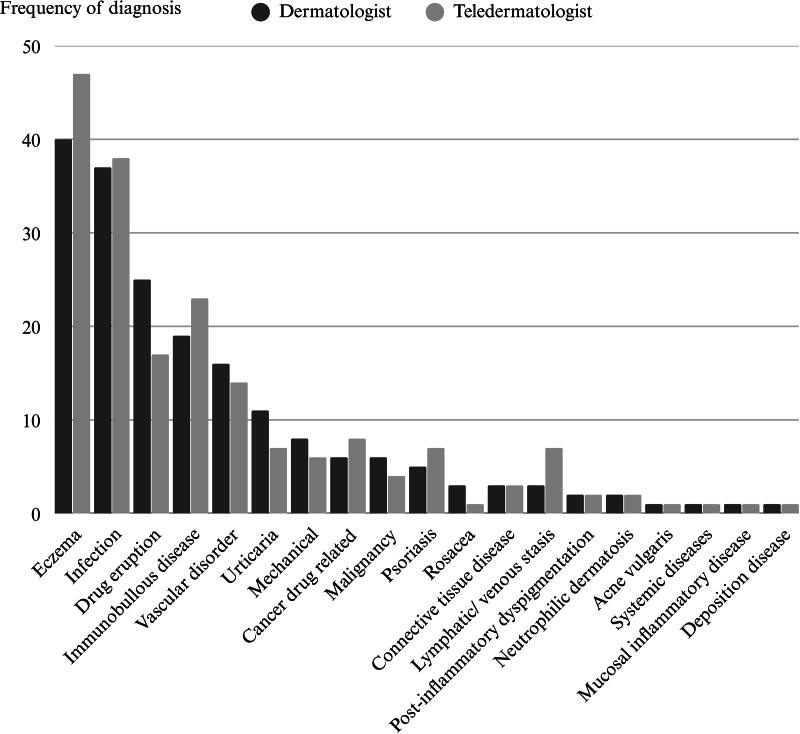
Preferred diagnosis of inpatient dermatologist and teledermatologist.

For the primary outcome of the study, the concordance between teledermatologists and inpatient dermatologists was 80% for the preferred diagnosis, with a kappa coefficient of 0.75 (95% confidence interval (CI) 0.71–0.78). Nineteen categories of dermatological diagnosis were identified (Fig. 2). When the preferred diagnosis was further specified according to those written in the questionnaire, there was a total number of 61 diagnoses (Supplementary Data S2). The concordance of this more specific diagnosis was 67.4%, with a kappa coefficient of 0.64 (95% CI 0.61–0.68). The concordance of differential diagnoses was 97.4% of the cases, with a kappa coefficient of 0.76 (95% CI 0.73–0.8) (Table 1).

**Table 1.** Agreement Between Teledermatologists and Inpatient Dermatologists

	Kappa coefficient (95% CI)	Percentage agreement (%)
Preferred diagnosis	0.75 (0.71–0.78)	80
Differential diagnoses	0.76 (0.73–0.8)	97.4
Specific diagnosis	0.64 (0.61–0.68)	67.4

CI, confidence interval.

### Secondary outcomes

For the secondary outcomes, the concordance of the diagnosis between the referring physicians and inpatient dermatologists (for cases with a specific referral diagnosis) was 56.2%, kappa coefficient 0.46 (95% CI 0.40–0.52), and between the referring physicians and the teledermatologist was 58.2%, kappa coefficient 0.51 (95% CI 0.46–0.57). The concordance for the decision of skin biopsy was 81.1%, kappa coefficient 0.55 (95% CI 0.48–0.62), whereas the concordance for the follow-up plan was lower with 65.8%, kappa coefficient 0.50 (95% CI 0.46–0.55). There was a higher rate of discharged patients without follow-up in the inpatient dermatologist arm (15.8%) than in the teledermatologist arm (4.7%).

The suggested biopsy rate of the teledermatologist was 31.02%, which was significantly different from that of the inpatient dermatologist (21.1%; *p* < 0.001). Skin biopsy was performed in 41 of the 190 (21.6%) patients. Of these 41 biopsies, a definitive diagnosis was confirmed by 24 of 41 (58.5%) for the teledermatologist group, whereas the percentage accuracy was 30/41 (73.2%) for the inpatient dermatologist. This comparison was made with the preferred diagnosis.

In addition, the final diagnosis was verified by various investigations, including blood for indirect immunofluorescent testing for anti-skin antibodies, skin scraping for fungus, skin scraping for scabies, and swab for polymerase chain reaction testing for herpes simplex virus. In total, 36 cases without skin biopsy results had the above investigation results. Of the 36 (63.9%) cases evaluated by inpatient dermatologists, 23 (63.9%) were confirmed by specific investigations. As for the teledermatologist, 22 of 36 (61.1%) cases were confirmed by these investigations. Overall, the total accuracy rate was 68.8% (53/77) for inpatient dermatologists and 59.7% (46/77) for the teledermatologist.

Factors affecting concordance were analyzed using a logistic regression model. Univariable analysis showed that the quality of the clinical photographs, biopsy, and follow-up plan agreement might be associated with the concordance result. These three items were analyzed using multivariable analysis. Better quality of clinical photos (either perfect or good) and agreement on the biopsy decision were independently associated with agreement of the preferred diagnosis (*p* < 0.001). Remarks stated in the survey of the teledermatologist often mentioned that the reason for fair to poor photo quality was due to incomplete photos not showing locations containing the rash for referral (Table 2).

**Table 2.** Univariable and Multivariable Analysis of Factors Associated with Agreement on Preferred Diagnosis Between Teledermatologist and Dermatologist

	Univariable analysis			Multivariable analysis		
	OR	95% CI	*p*	OR	95% CI	*p*
Age	0.93	0.07–1.01	0.45			
Gender	1.00	0.49–2.04	1.00			
Race	1.00	0.11–9.21	1.00			
Referral from Medicine	1.41	0.64–3.14	0.40			
Referral with a specific diagnosis	1.46	0.71–3.01	0.31			
Biopsy done	0.60	0.27–1.35	0.22			
Quality of clinical photo(perfect or good versus fair or unacceptable)	5.34	2.49–11.43	<0.001	5.44	2.48–11.91	<0.001
Follow-up plan agreement	1.53	0.74–3.17	<0.001	1.23	0.54–2.81	0.62
Biopsy plan agreement	2.92	1.31–6.52	<0.001	2.82	1.14–7.01	0.03

CI, confidence interval; OR, odds ratio.

Moreover, as shown in Table 3, for patients referred for ulcers, eczema, psoriasis, and nodules, both the teledermatologists and the inpatient dermatologists completely agreed with each other’s diagnosis.

**Table 3.** Parameters Associated with the Agreement on Preferred Diagnosis

	Total	Agree on preferred diagnosis	Disagree on preferred diagnosis	*p*-Value
	Mean (SD)	Mean (SD)	Mean (SD)	
	*N* (%)	*N* (%)	*N* (%)	
Age	64.11 (17.44)	64.59 (18.05)	62.21 (14.82)	0.32
Gender				0.57
Male	95 (50)	76 (50.0)	19 (50.0)	
Female	95 (50)	76 (50.0)	19 (50.0)	
Race				0.77
Chinese Asian	185 (97.5)	148 (97.0)	37 (97.4)	
Others	5 (2.5)	4 (3.0)	1 (2.6)	
Biopsy				0.22
Biopsy done	41 (21.6)	30 (19.7)	11 (29.9)	
No biopsy done	149 (78.4)	122 (80.3)	27 (71.1)	
Referral ward				0.26
Medical	145 (76.3)	118 (77.6)	27 (71.1)	
Nonmedical	45 (23.7)	34 (22.4)	11 (28.9)	
Referral ward in categories				0.88
Medicine	114 (60)	92 (60.5)	22 (57.9)	
Medicine (specialty)	31 (16.3)	26 (17.1)	5 (13.2)	
Orthopedics	23 (12.1)	19 (12.5)	4 (10.5)	
Surgery	14 (7.4)	10 (6.6)	4 (10.5)	
Psychiatry	2 (1.1)	1 (0.7)	1 (2.6)	
Clinical oncology	5 (2.6)	4 (2.6)	1 (2.6)	
Obstetrics and gynecology	1 (5.3)	0 (0.0)	1 (2.6)	
nitial reasons for consultation				0.20
With no specific diagnosis	101 (53.2)	78 (77.2)	23 (22.8)	
Rash	57 (30)	46 (30.3)	11 (28.0)	0.34
Blister	10 (5.3)	8 (5.3)	2 (5.3)	0.10
Dermatitis	7 (3.7)	4 (2.6)	3 (7.9)	1.00
Ulcers	6 (3.2)	6 (3.9)	0 (0.0)	0.03
Urticarial eruptions	5 (2.6)	3 (2.0)	2 (5.3)	1.00
Nodules	4 (2.1)	4 (2.6)	0 (0.0)	0.12
Pustules	3 (1.6)	2 (1.3)	1 (2.6)	1.00
Dyspigmentation	2 (1.1)	2 (1.3)	0 (0.0)	0.50
Mucosal lesions	3 (1.6)	1 (0.7)	2 (5.3)	1.00
Purpuras	1 (0.5)	1 (0.7)	0 (0.0)	
Not specified	3 (1.8)	1 (0.7)	2 (5.3)	
With a specific diagnosis	89 (46.8)	74 (83.1)	15 (16.9)	
Infection	33 (17.4)	27 (17.8)	6 (15.8)	0.48
Immunobullous disease	14 (7.4)	12 (7.9)	2 (5.3)	0.49
Eczema	8 (4.2)	8 (5.3)	0 (0.0)	0.007
Drug eruption	7 (3.7)	6 (3.9)	1 (2.6)	0.12
Psoriasis	6 (3.2)	6 (3.9)	0 (0.0)	0.03
Annular erythema	5 (2.6)	4 (2.6)	1 (2.6)	0.37
Cancer therapy related eruptions	5 (1.6)	3 (2.0)	2 (5.3)	1.00
Vascular lesions	4 (2.1)	3 (2.0)	1 (2.6)	0.62
Malignancies	2 (1.1)	2 (1.3)	0 (0.0)	0.50
Neutrophilic dermatosis	2 (1.1)	1 (0.7)	1 (2.6)	1.00
Rosacea	2 (1.1)	1 (0.7)	1 (2.6)	1.00
Mechanical eruptions	1 (0.5)	1 (0.7)	0 (0.0)	
Quality of clinical photos provided				<0.001
Perfect	7 (3.7)	7 (4.6)	0 (0.0)	
Good	117 (61.6)	104 (68.4)	13 (34.2)	
Fair	57 (30.0)	37 (24.3)	20 (52.6)	
Unacceptable	8 (4.2)	3 (2.0)	5 (62.5)	
Follow up plan agreement				0.17
Agree	125 (65.8)	103 (67.8)	22 (57.9)	
Disagree	65 (34.2)	49 (32.2)	16 (42.1)	
Biopsy recommendation				0.009
Agree	154 (81.1)	129 (84.9)	25 (65.8)	
Disagree	36 (18.9)	23 (15.1)	13 (34.2)	
Total	190 (100)	152 (80.0)	38 (20.0)	

SD, standard deviation.

## Discussion

The results of this study revealed substantial agreement between the teledermatologist and the inpatient dermatologist regarding the preferred diagnosis (80%, kappa coefficient 0.75). When the other two differential diagnoses were considered, the concordance increased further (97.4%; kappa coefficient, 0.76). These findings demonstrate the reliability of a practical and real-world inpatient teledermatology consultation service in Hong Kong.

Compared with other similar studies, our study showed better performance by a teledermatologist. These studies also examined the concordance of skin biopsy recommendations, and the results were similar to those of the present study. Although we achieved a concordance of 81% for biopsy decisions, the biopsy rate (30.5%) was higher in the teledermatology setting. Similarly, the biopsy rate was higher in the teledermatologist group in other studies.^13,16,17^ This could be explained by the lower certainty in assessing patients using the store-and-forward images. One might argue that this may lead to unnecessary biopsy. However, a consistently higher skin biopsy rate could also enhance patient safety by improving diagnostic accuracy. Furthermore, when compared with a non-telemedicine study, a similar biopsy rate of 31% for face-to-face consultations was observed.^18^

The follow-up plan or triage decision is crucial in evaluating the reliability of a teledermatology program. Nevertheless, as shown in other studies, the concordance of the follow-up plans was less substantial. Teledermatologists suggested more inpatient monitoring and discharged fewer patients without follow-up, which could be attributed to a more cautious approach when patients were assessed through telemedicine. This difference could compensate for the slightly lower telemedicine accuracy. The reliability of teledermatology to triage inpatient consultations was also demonstrated in a US study.^17^

For the in-house dermatologist in our study, the 68.8% accuracy of diagnosis was comparable with another study on inpatient dermatology,^18^ implying the validity of the comparison in our study. The accuracy of inpatient dermatologists was slightly higher than that of the teledermatologists, which aligns with the findings of a previous outpatient teledermatology study.^7^

Our study proved that the reliability of teledermatology is related to the quality of referral images. This was affected by the individuals who captured the images, which could be taken by patients, clinicians, other health care providers, or medical students. High-resolution cameras, mobile devices, and smartphones can all be used to obtain store-and-forward images, and have been studied.^19^ In general, clinician-obtained images yield higher diagnostic agreement.^9^ In this study, we used a pragmatic approach to simulate a real-world setting. Because sophisticated cameras for clinical photos may not always be available in clinical practice, we utilized existing facilities. Apple^TM^ iPads with security logins were available in every ward, and either nurses or the attending physician could take clinical photographs of the patients. This approach resulted in substantial reliability.

The sample size was larger than that used in other similar studies, thus enhancing the reliability of the study. We also demonstrated a significant association between the quality of clinical photographs and concordance results, suggesting that a photo-taking protocol could potentially improve the reliability of inpatient teledermatology.

This study also serves as a basis for multispecialty hospitals lacking in-house dermatologists to implement inpatient teledermatology programs to enhance specialist accessibility. A study in the United States revealed that only 51% of patients could receive dermatology input despite being admitted for a skin condition.^11^ In Hong Kong, only two public hospitals have in-house dermatologists. The practice of inpatient teledermatology in our locality has continued beyond the current study period and has been effective in increasing access to dermatology care.

This study had some limitations. Only two qualified dermatologists were available to assess the stored clinical photographs. Moreover, they did not assess the same cases, which was the design of other teledermatology studies. Therefore, we had to consider the inter-rater difference, which could partially explain the discordance. The lack of teledermatology training and experience in Hong Kong may also affect the diagnostic reliability of teledermatologists. However, years of experience in inpatient dermatology should have compensated for this.

In addition, this was a single-center study conducted on a local population; therefore, the study results might not be generalizable to other ethnicities. However, they would be applicable to hospitals with similar settings to those of Chinese Asians.

Nevertheless, conducting teledermatology studies can be challenging, as a gold-standard diagnosis may not be available for many dermatological diseases. When the two groups disagreed with each other, it was uncertain which diagnosis was correct. Often, the response to treatment and disease progression must be monitored to determine the ultimate diagnosis. Therefore, accuracy analysis is subject to some errors.

Patient outcomes and cost-effectiveness were not assessed in the present study. Future research could include non-inferior randomized controlled trials to examine the outcomes of patients receiving traditional care versus those under a teledermatology program. Outcomes could include the time to healing of lesions, length of hospital stay, degree of symptomatic relief, and other patient-reported outcomes.

Although the concordance was substantial, there was still a small number of patients with a disagreed diagnosis. Without a face-to-face physical examination, a complete skin examination might not be performed.^20^ Determining whether the primary lesion was raised, for example, papular or nodular, would alter possible differential diagnoses. Therefore, good lighting and a high-resolution display screen are required to show the three-dimensional quality of the referred photo.

Overall, we have demonstrated a reliable and accurate inpatient teledermatology program in a real-world setting for Asian patients. Its utilization should be encouraged to enhance specialist accessibility and, hence, improve patient management.

## Supplementary Material

Supplementary Data S1

Supplementary Data S2

## Data Availability

Data and material available upon request.

## Abbreviations Used

CI: Confidence interval
COVID-19: Coronavirus disease 2019
HA: Hospital authority
HKU: The University of Hong Kong
HKW: Hong Kong West Cluster
HSV: Herpes Simplex Virus
IRB: Institutional Review Board
OR: Odds ratio
PCR: Polymerase chain reaction
SD: Standard deviation
