# Supplementary material for: Reliability and Accuracy of Inpatient Teledermatology in Asian Patients
Source: Telemed Rep. 2025 Sep 17;6(1):259–67. doi: 10.1177/26924366251380372 (PMC12543428; doi:10.1177/26924366251380372)
Supplement: Supplementary Data S1 [file 26924366251380372_suppl_datas1.pdf]

Supplemental file 1

Dermatologist and Teledermatologist Questionnaire

Dermatologist code: ______________________ Date: ______________________

I evaluated this patient
- ☐ In person, face to face
- ☐ Via teledermatology only

1. What are your top three differential diagnoses?

   ____

   ____

   ____

2. Which of these is your preferred diagnosis?

   ____

3. Do you recommend a skin biopsy? ☐ Yes ☐ No

4. What will be your FU plan?
   - ☐ No FU
   - ☐ FU in general clinic
   - ☐ FU in dermatology clinic
   - ☐ In-patient monitoring

5. Please rate the quality of the clinical photos taken
   - ☐ Perfect
   - ☐ Good
   - ☐ Fair
   - ☐ Unacceptable

Any comment?
