# Supplementary material for: Reliability and Accuracy of Inpatient Teledermatology in Asian Patients
Source: Telemed Rep. 2025 Sep 17;6(1):259–67. doi: 10.1177/26924366251380372 (PMC12543428; doi:10.1177/26924366251380372)
Supplement: Supplementary Data S2 [file 26924366251380372_suppl_datas2.pdf]

Supplemental file 2

Descriptive statistics of specific diagnoses		
	n (%)	
	**Teledermatologist**	**Dermatologist**
Acneiform eruption	4 (2.11)	3 (1.58)
Acne vulgaris	1 (.53)	1 (.53)
Acute urticaria	6 (3.16)	9 (4.74)
Adult onset stills disease	1 (.53)	1 (.53)
Allergic contact dermatitis	7 (3.68)	9 (4.74)
Aphthous ulcer	0 (.00)	1 (.53)
Arterial ulcer	0 (.00)	3 (1.58)
Asteatotic eczema	7 (3.68)	7 (3.68)
Atopic eczema	1 (.53)	3 (1.58)
Bacterial cellulitis	10 (5.26)	11 (5.79)
Behcets disease	1 (.53)	0 (.00)
Bullous pemphigoid	20 (10.53)	19 (10.00)
Calciphylasix	2 (1.05)	0 (.00)
Cancer therapy related	1 (.53)	0 (.00)
Chemotherapy extravasation	1 (.53)	1 (.53)
Deposition disease	1 (.53)	1 (.53)
Dermatomyositis	0 (.00)	1 (.53)
Diabetic dermopathy	0 (.00)	1 (.53)
Discoid eczema	0 (.00)	3 (1.58)
DRESS syndrome	2 (1.05)	2 (1.05)
Drug eruption	0 (.00)	1 (.53)
Eczema	22 (11.58)	4 (2.11)
Edema blister	6 (3.16)	6 (3.16)
Embolic phenomenon	0 (.00)	1 (.53)
Erythema annulare centrifugum	1 (.53)	1 (.53)
Erythema multiforme	0 (.00)	1 (.53)
Erythrodermic psoriasis	0 (.00)	1 (.53)
Friction blister	0 (.00)	1 (.53)
Hand foot syndrome	0 (.00)	1 (.53)
Herpes simplex	6 (3.16)	5 (2.63)
Immune related adverse event	1 (.53)	1 (.53)
Irritant contact dermatitis	1 (.53)	0 (.00)
Leukaemia cutis	1 (.53)	1 (.53)

Livedoid vasculopathy	0 (.00)	2 (1.05)
Lymphedema complications	2 (1.05)	1 (.53)
Lymphoma cutis	2 (1.05)	4 (2.11)
Maculopapular drug eruption	16 (8.42)	21 (11.05)
Mycobacterium infection	2 (1.05)	2 (1.05)
Oral lichen planus	1 (.53)	0 (.00)
Paronychia	1 (.53)	0 (.00)
Petechiae	3 (1.58)	4 (2.11)
Pemphigus vulgaris/ foliaceus	2 (1.05)	0 (.00)
Photodermatitis	1 (.53)	1 (.53)
Plaque psoriasis	6 (3.16)	3 (1.58)
Post inflammatory hyperpigmentation	2 (1.05)	2 (1.05)
Pustular psoriasis	1 (.53)	1 (.53)
Pyoderma gangrenosum	2 (1.05)	2 (1.05)
Rosacea	1 (.53)	3 (1.58)
Scabies	1 (.53)	2 (1.05)
SDRIFE	0 (.00)	1 (.53)
Seborrheic dermatitis	1 (.53)	2 (1.05)
Squamous cell carcinoma	1 (.53)	1 (.53)
Stasis eczema	8 (4.21)	12 (6.32)
Subacute cutaneous lupus erythematosus	1 (.53)	1 (.53)
Thrombophlebitis	1 (.53)	1 (.53)
Tinea	7 (3.68)	8 (4.21)
Uraemic pruritis	1 (.53)	0 (.00)
Vasculitis	8 (4.21)	5 (2.63)
Venous ulcer	4 (2.11)	2 (1.05)
Viral exanthem	11 (5.79)	8 (4.21)
Zoster	1 (.53)	1 (.53)
